# Esophageal Squamous Papillomatosis Associated Low-Grade Dysplasia Treated with Endoscopic Submucosal Dissection

**DOI:** 10.5152/tjg.2024.24178

**Published:** 2024-09-01

**Authors:** Abdullah Murat Buyruk, Çağdaş Erdoğan

**Affiliations:** 1 Department of Gastroenterology, Ege University School of Medicine, İzmir, Türkiye; 2 Department of Gastroenterology, University of Health Sciences, Ankara Etlik City Hospital, Ankara, Türkiye

Dear Editor,

We wish to bring to your attention a noteworthy case concerning the management of esophageal squamous papillomatosis (ESPs) with associated low-grade dysplasia, treated successfully using endoscopic submucosal dissection (ESD). Esophageal squamous papilloma (ESP) is a rare (incidence = 0.01%-0.45%) benign epithelial tumor of the esophagus, and it is usually detected incidentally and asymptomatic.^1^ Tumor size is typically 2-8 mm. Esophageal squamous papillomatosis (ESPs) is an extremely rare pathology that occurs when multiple papillomata come together and is predominantly symptomatic.^2^ Esophageal squamous papillomatosis may present in many clinical manifestations, such as symptomatic dysphagia, anemia, and hematemesis.^3^ Although the possibility of malignant transformation in ESP/ESPs has been demonstrated in a small number of cases, no defined risk factors for malignant transformation have been reported thus far. Both the possibility of malignant transformation and the fact that patients are mostly symptomatic highlight the need for treatment in these patients. However, there is no clear consensus on the treatment of these patients. Endoscopic submucosal dissection (ESD) is the first-line endoscopic treatment method recommended by the guidelines as it allows en bloc resection of large superficial epithelial tumors of the esophagus and facilitates accurate histopathological examination. To the best of our knowledge, no study in the literature has examined the suitability and safety of the ESD method in ESPs treatment. In the current report, we present a case of broad-based ESPs with dysphagia who was treated with ESD.

A 66-year-old female patient presented to our clinic with dysphagia to solids and liquids for the last year. The patient was on valsartan/hydrochlorothiazide for arterial hypertension. She had no history of smoking or alcohol use. The patient’s blood tests were checked for human papillomavirus (HPV), but the tests were negative in this regard. No chronic inflammatory process was detected in the patient’s examinations. The patient did not have symptoms of gastroesophageal reflux. Esophagogastroduodenoscopy (EGD) showed a flat area with wart-like protrusions in the middle third of the esophagus (25-31 cm from incisors) that the lesion involved 60% of the esophageal lumen (Figure 1). The application of Lugol’s iodine solution stains the normal squamous epithelium dark brown, whereas dysplastic or neoplastic areas appear as unstained or light yellow due to the lack of glycogen in the cells. This area was stained and subsequently observed via Lugol chromoendoscopy (Figure 1). Histopathology of the biopsy was consistent with ESPs. The radial echoendoscope (Olympus) showed that ESPs were confined to the mucosa layer (Figure 1). The settings for this examination included a frequency of 7.5 MHz, a depth of 5 cm, and the use of a balloon for optimal contact with the mucosal surface. Due to the presence of dysphagia and the likelihood of dysplasia associated with ESPs, we obtained informed consent from the patient. We removed the ESPs en-bloc (lesion size: 55 × 36 mm) through ESD during ESD, a procedure lasting approximately 55 minutes (Figure 1). For the ESD procedure, Olympus DualKnife (KD-650L/Q) and HookKnife (KD-620LR) were used.

The lesion had irregular borders (Figure 3). Histopathologic examination revealed papillomatous projections with fibrovascular cores and free-floating papillary fragments. Some parts of the lesion showed basal cell hyperplasia and low-grade dysplasia accompanied by rare koilocytosis. Ki-67 was restricted to the proliferative layer, and p16, which was supposed to show strong positivity in HPV-related tumors, was negative immunohistochemically. The patient was re-presented to our clinic 2 months after ESD due to dysphagia, and follow-up EGD showed a stricture at the ESD site (Figure 2). The stricture was treated with balloon dilatation. By the first month after balloon dilatation, it was observed that the dysphagia had almost completely resolved. Follow-up EGD at 6 months showed no recurrence (Figure 3).

To the best of our knowledge, fewer than 20 cases have been reported thus far.^3^ ESPs is an extremely rare esophageal tumor thought to be benign. Esophageal squamous papillomatosis seem more common in middle-aged male individuals in Western countries, but in Asian countries, women are more commonly diagnosed with ESP than men. Esophageal squamous papillomatosis are commonly found in the middle and distal thirds of the esophagus, although they have been found in all parts of the esophagus.

The etiology of Esophageal Squamous Papilloma (ESP) is not fully understood, with both a hyper-regenerative response from factors like gastroesophageal reflux disease, alcohol consumption, smoking, and Human Papillomavirus (HPV) infection considered primary influences. The association between HPV, particularly high-risk strains, and the development of ESP and squamous cell carcinoma (SCC) is an area of ongoing research. Despite varying HPV detection rates in ESP, the direct identification of HPV in cases of ESP with malignant transformation has proven challenging. Reports have indicated a significant risk of malignant transformation within ESPs, with both SCC and adenocarcinoma identified among documented cases. This underscores the importance of early detection and intervention, particularly in the precancerous stages of ESPs.

Diagnostically, ESP is characterized endoscopically by wart-like projections and exophytic growth, with techniques such as narrow-band imaging (NBI) and Lugol chromoendoscopy aiding in differentiating ESPs from SCC. Lugol chromoendoscopy has been valuable in assessing the malignant potential within ESPs.

Endoscopic submucosal dissection (ESD) is highlighted as a preferred treatment for superficial epithelial tumors of the esophagus, including ESPs, despite the lack of specific treatment guidelines.^4,5^ ESD’s minimally invasive nature, compared to traditional surgical resection, offers advantages such as enabling comprehensive histopathological examination and the potential for complete lesion removal. However, ESD is technically demanding and associated with a higher risk of complications, such as stricture formation following extensive resections. Effective management strategies, including post-ESD stricture treatment with balloon dilatation, are critical for patient recovery and highlight the need for advancements in preventive measures against such complications.

In conclusion, ESPs is a rare benign tumor of the esophagus but has a risk of malignant transformation. In cases with ESPs, treatment should be strongly recommended in the presence of persistent symptoms and suspicion of dysplasia. As demonstrated by the case presented in this report, ESD can be an effective and reliable option in the treatment of large ESPs.

## Data Availability Statement

The datasets used and/or analyzed during the current study are available from the corresponding author on reasonable request.

## Figures and Tables

**Figure 1.**
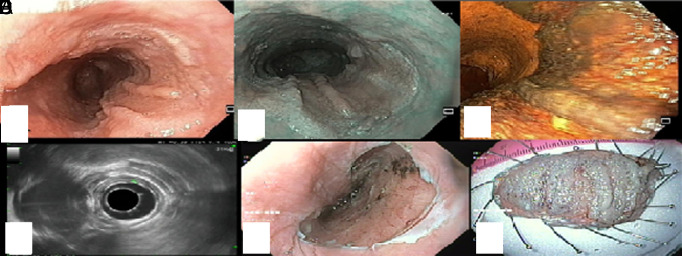
White light endoscopy shows a raised mucosal area with wart-like protrusions in the middle esophagus (A) With Narrow Band Imaging, wart-like protrusions on the raised area of the mucosa are observed as more prominent (B). Raised area from the mucosa stains in chromoendoscopy with Lugol dye (C); Defined area in the radial echoendoscope is limited to the mucosa (D). Esophagogastroduodenoscopy (EGD) shows the area with esophageal squamous papillomatosis that was resected by the endoscopic submucosal dissection (ESD) method (E). The specimen after en-bloc resection of the esophageal squamous papillomatosis area with ESD (F). EGD, esophagogastroduodenoscopy; ESD, endoscopic submucosal dissection.

**Figure 2.**
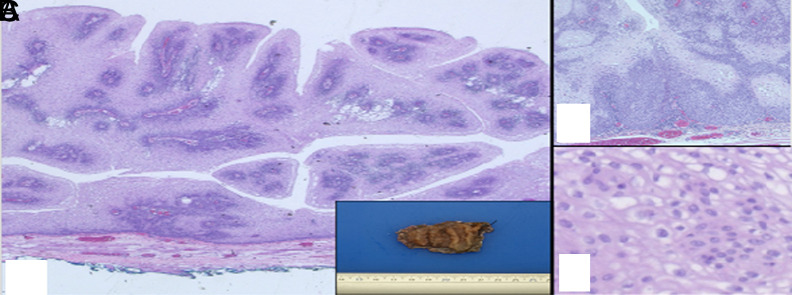
The characteristics papillary projections of the lesion (hematoxylin–eosin, ×2.5) Inset: Macroscopic appearance of the lesion with irregular borders (A); basal cell hyperplasia and focal dysplasia (hematoxylin–eosin, ×10) (B); mild atypia consistent with low-grade dysplasia and koilocytosis (hematoxylin–eosin, ×20) (C).

**Figure 3.**
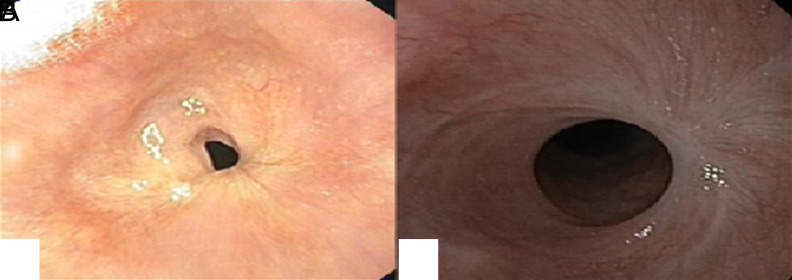
Control EGD shows stenosis in the endoscopic submucosal dissection (ESD) area (A). The appearance of the ESD scar at 6 months (B).

## References

[1] Caroline D’huart M Chevaux JB Marchal Bressenot A , et al. Prevalence of Esophageal Squamous Papilloma (ESP) and Associated Cancer in Northeastern France. Accessed 2023 May 15. Available at: http://dx.doi.org/. 10.1055/s-0034-1390976 PMC4477031 26135647

[2] Attila T Fu A Gopinath N Streutker CJ Marcon NE . Esophageal papillomatosis complicated by squamous cell carcinoma. Can J Gastroenterol. 2009;23(6):415 419. (10.1155/2009/659820) 19543571 PMC2721808

[3] Cho JY Cheung DY Kim TJ Kim JK . Case report a case of esophageal squamous cell carcinoma in situ arising from esophageal squamous papilloma. Clin Endosc. 2019;52(1):72 75. (10.5946/ce.2018.058) 30021250 PMC6370924

[4] Ishihara R Arima M Iizuka T , et al. Japan Gastroenterological Endoscopy Society Guidelines Committee of ESD/EMR for Esophageal Cancer. Endoscopic submucosal dissection/endoscopic mucosal resection guidelines for esophageal cancer. Dig Endosc. 2020;32(4):452 493. (10.1111/den.13654) 32072683

[5] Guo HM Zhang XQ Chen M Huang SL Zou XP . Endoscopic submucosal dissection vs endoscopic mucosal resection for superficial esophageal cancer. World J Gastroenterol WJG. 2014;20(18):5540 5547. (10.3748/wjg.v20.i18.5540) 24833885 PMC4017070

